# Initial Sliding Wear Kinetics of Two Types of Glass Ionomer Cement: A Tribological Study

**DOI:** 10.1155/2014/790572

**Published:** 2014-06-29

**Authors:** Cyril Villat, Pierre Ponthiaux, Nelly Pradelle-Plasse, Brigitte Grosgogeat, Pierre Colon

**Affiliations:** ^1^UFR d'Odontologie, Université de Lyon, 11 rue Guillame Paradin, 69372 Lyon, France; ^2^Service de Consultations et Traitements Dentaires, Hospices Civils de Lyon, 6-8 Place Deperet, 69365 Lyon, France; ^3^Laboratoire des Multimatériaux et Interfaces, CNRS UMR 5615, Université de Lyon, 11 rue Guillaume Paradin, 69372 Lyon, France; ^4^Laboratoire de Génie des Procédés et Matériaux, Ecole Centrale Paris, Chemin des Vignes, 92295 Châtenay-Malabry, France; ^5^UFR d'Odontologie, Université Denis Diderot Paris 7, 5 rue Garancière, 75006 Paris, France

## Abstract

The aim of this work was to characterize the initial wear kinetics of two different types of glass ionomer cement used in dentistry (the conventional glass ionomer cement and the resin-modified glass ionomer cement) under sliding friction after 28-day storing in distilled water or Ringer's solution. Sliding friction was applied through a pin-on-disk tribometer, in sphere-on-plane contact conditions, under 5 N normal load and 120 rotations per minute. The test lasted 7500 cycles and replicas were performed at 2500, 5000 and 7500 cycles. A profilometer was used to evaluate the wear volume. Data were analysed using Student's *t*-test at a significant level of 5%. There is no statistical significant difference between the results obtained for a given material with the maturation media (*P* > 0.05). However, for a given maturation medium, there are significant statistical differences between the data obtained for the two materials at each measurement (*P* < 0.0001). The wear rates of both materials decrease continuously during the running-in period between 0 and 2500 cycles. After 2500 cycles, the wear rate becomes constant and equal for both materials. The resin matrix contained in the resin-modified glass ionomer cement weakens the tribological behaviour of this material.

## 1. Introduction

Wear of dental materials has consequences both systemic and biological that require precise understanding of the tribological behaviour of such materials [1]. However, wear of dental materials is a complex phenomenon that involves different parameters [1–4]:

- nature, properties of the antagonist counterbody (hardness, shape, composition,…, etc.), roughness, and porosity of the tested material;
- antagonist contacting surfaces;
- type of the used wear testing device, characteristics of the relative motion, applied normal load, sliding speed, and total sliding distance;
- environmental conditions (temperature, composition, and pH of the surrounding medium);
- presence of a lubricant or of a third body in the contact interface.

Two types of wear testing devices have been described in the literature for evaluating the wear of dental materials: the two-body abrasion device (direct contact surfaces) and the three-body abrasion device (interposition of a third body between the two surfaces). The first characterizes non-masticatory tooth movement like bruxism and attrition [3]. The second one characterizes masticatory tooth movements [2, 3]. Nevertheless, three-body wear can occur in two-body devices when particles are derived from the restoration due to wear fatigue [4].

To evaluate wear, most studies use the measurement of wear depth; however, measurement of volume seems to be more appropriate [5]. In fact, there is a relationship between depth and lost volume [1, 6], but it depends on the shape of the contacting surfaces.

Few results are available concerning the wear kinetics of dental materials and particularly of glass ionomer cement. The purpose of most papers concerns more the “material ranking” than the kinetic and mechanical laws governing this process [4, 6–11]. Wear kinetics data are given for various restoration materials (unfortunately, not GICs) by DeLong [5], who reported linear relationships between the worn volumes and time, under cyclic rubbing conditions applied with an “artificial mouth chewing machine.” This result suggests that the tested materials have a homogeneous behaviour in accordance with the Archard's law [12].

The glass ionomer cement is widely used in many medical domains and particularly in restorative dentistry. It is much susceptible than composite resins to the chemical properties of the surrounding medium which may react with the hydrogel matrix and affect the setting process [13]. It is most indicated for restorative procedures for temporary restorations in permanent denture or permanent restorations in temporary denture. It has been shown that wear behaviour of glass ionomer cement depends on several parameters such as polymer matrix, glass fillers, and the interfacial bonding between glass and matrix [7]. The setting process of glass ionomer cement can be considered as completed after 4 weeks, taking as a criterion that the microhardness has reached a stationary value [14]. In fact, the setting reaction could be divided in 3 steps: ion extraction from glass particles between 3 and 7 minutes, cross-linking of polyacrylic acid molecules with Al^3+^ between 2 and 5 days [14, 15], and then increase of mechanical properties (e.g., microhardness) which extends for a longer time [14, 16, 17].

The purpose of this study was to consider the effect of two maturation media (distilled water and Ringer's solution where the salts could interact with the material) on the wear resistance and to describe the early sliding wear kinetics of mature samples of the high viscosity conventional glass ionomer cement (C-GIC) and of the resin-modified glass ionomer cement (RM-GIC).

## 2. Materials and Methods

### 2.1. Tribological Experiment

The high viscosity conventional glass ionomer cement (Fuji IX GP Fast Capsule, GC Corp., Tokyo, Japan) and the resin-modified glass ionomer cement (Fuji II LC Capsule, GC Corp., Tokyo, Japan) were tested.

Twenty-four samples of each type of the tested cement were performed in hexagonal molds (8 mm diameter and 2 mm high). RM-GIC samples were photopolymerized for 60 seconds using a halogen light curing unit (Astralis 7, mode HIP: 750 mW/cm², Ivoclar-Vivadent, Schaan, Lichtenstein). The samples were polished with an abrasive paper #1200. Then, the samples were immersed in 6 mL aqueous solution and stored in a climatic chamber at 37°C during 28 days (SH 340, Secasi Technologies, Pessac, France). Twelve samples of C-GIC and twelve samples of RM-GIC were immersed in distilled water. Twelve samples of C-GIC and twelve samples of RM-GIC were stored in Ringer solution.

**Figure 1:**
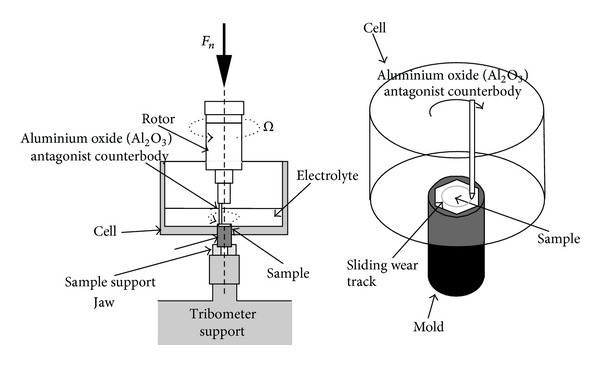
Schema of the wear device.

Samples mounted in the hexagonal molds were fixed at the bottom of a PVC cell containing 150 mL Ringer solution with the working surface facing upwards. The cell was mounted on a pin-on-disc tribometer, and sliding friction was applied by rotating the alumina cylinder pin with spherical end (diameter of the spherical end: 200 mm) against the sample surface (Figure 1).

The normal load applied during the tests was 5 N. A rotation frequency of 2 Hz was applied in all tests. The radius of the wear track was set at 2.5 mm, giving sliding speed of 31.4 mm s^−1^. The total number of cycles in a test was 7500.

**Figure 2:**
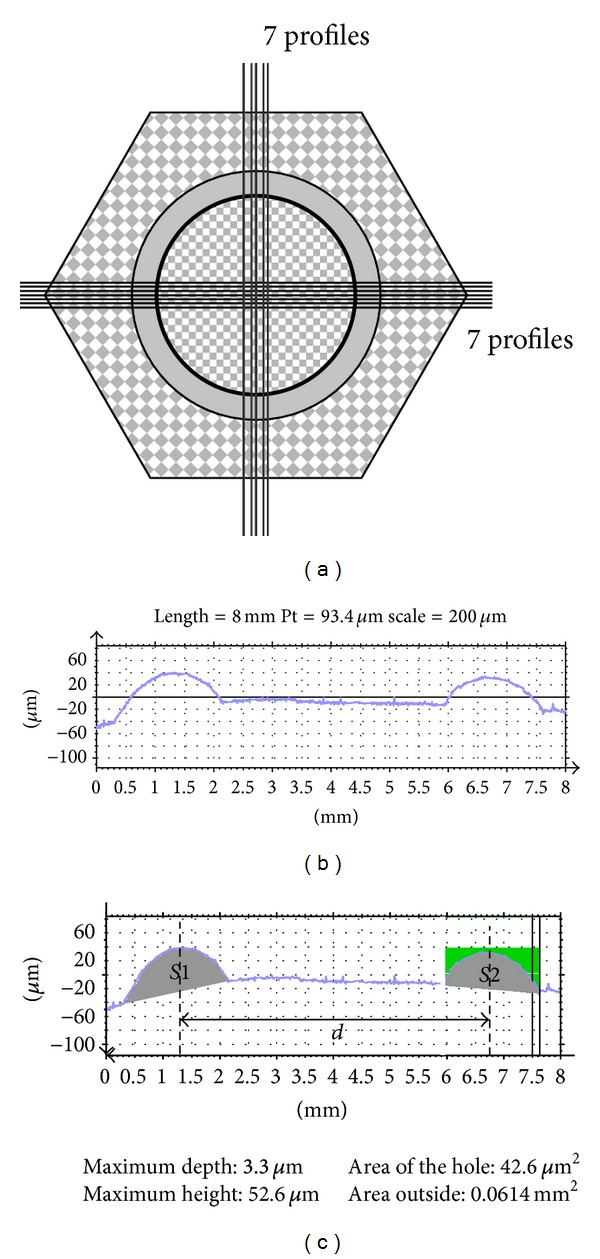
(a) Profile extraction, (b) profile extracted after removal of the defects, (c) diameter measurement (*d*), and surface measurement (*S*1 and *S*2).

Silicone replicas of the worn surface (Aquasil Ultra LV Regular Set, Dentsply DeTrey Gmbh, Konstanz, Germany) were performed at 0, 2500, 5000, and 7500 cycles. They were metallized with gold, and then 14 cross-sectional profiles of the imprint were surveyed in two groups of 7 along two perpendicular diameters of the track on each replica (Figure 2(a)), with a high resolution optical profilometer (Station Micromesure STIL, France; lateral resolution 1 *μ*m and vertical resolution 100 nm).

### 2.2. Measurements

Profilometry data were analyzed with specific software (Mountains Map Universal, Digital Surf, Besançon, France). For each replica, the 28 cross-section areas *S*_*i*_ (*i* = 1,2,…, 28) of the wear track were measured (Figures 2(b) and 2(c)), and a worn volume *W*_*i*_ was calculated for each area according to the following expression:

(1) $$\begin{array}{c} W_{i}=\pi \;ds_{i}, \end{array}$$

where *d* is the mean diameter of the wear track (*d* = 5.0 mm) and *S*_*i*_ the surface of the wear track for each section.

The average worn volume is then calculated for each replica according to the following formula:

(2) $$\begin{array}{c} W=\frac{1}{28}\overset{28}{\underset{i=1}{\sum }}W_{i}. \end{array}$$

The worn volume has then been divided by the applied load to obtain a normalized worn volume: *W*_*n*_ = 1/5*W*.

### 2.3. Statistical Analysis

Data were analysed using Student's *t*-test at a significant level of 5% (*P* < 0.05) with StatView software (StatView, SAS Institute Inc., Cary, NC, USA).

## 3. Results

**Figure 3:**
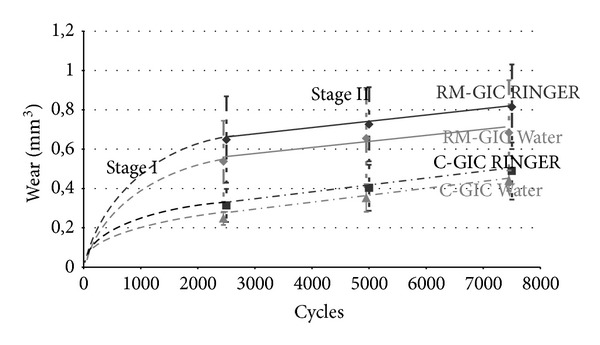
Wear kinetics in regard to the immersion media. (Curves have been performed according to the mean values (for Stage II). Stage I is represented as a dotted curve between 0 and 2500 cycles for each group. Vertical dotted lines represent the standard deviations).

The kinetics wear profiles are presented in Figure 3. After 2500, 5000, and 7500 cycles, there is no statistical significant difference between the results obtained with different maturation media, distilled water, and Ringer solution (*P* > 0.05). However, for a given medium, there are significant statistical differences between the data obtained with the two materials at these three values of the number of cycles (*P* < 0.0001). For both materials and both media, Figure 3 shows similar evolutions of wear with the number of cycles. A linear increase is observed between 2500 and 7500 cycles with close wear rates for each pair material/immersion media.

Variations of wear with time were analyzed using linear regression. The results are shown in Table 1. The average wear rates for each material and each maturation medium are shown in Table 2.

The wear rates and the normalized wear rates are as follows.

- For C-GIC matured in distilled water, *W* = (38 ± 29) · 10^−6^ mm^3^/cycle and *W*_*n*_ = (4.8 ± 3.7) · 10^−7^ mm^3^/N*·*mm.
- For C-GIC matured in Ringer's solution, *W* = (35 ± 12) · 10^−6^ mm^3^/cycle and *W*_*n*_ = (4.5 ± 1.5) · 10^−7^ mm^3^/N*·*mm.
- For RM-GIC matured in distilled water, *W* = (29 ± 9) · 10^−6^ mm^3^/cycle and *W*_*n*_ = (3.7 ± 1.1) · 10^−7^ mm^3^/N*·*mm.
- For RM-GIC matured in Ringer's solution, *W* = (33 ± 19) · 10^−6^ mm^3^/cycle and *W*_*n*_ = (4.2 ± 2.4) · 10^−7^ mm^3^/N*·*mm.

No data are available before 2500 cycles, but the fact that the lines drawn between 2500 and 7500 cycles intersect the *y*-axis at positive values indicates that between 0 and 2500 cycles the wear rate was higher than the constant value obtained between 2500 and 7500 cycles.

In Figure 3, the variation of wear in the interval 0–2500 cycles is schematically represented by the dotted parts of the curves. Consequently, two stages can be identified in the evolution of wear during the present study:

- the first stage (Stage I) with high initial wear rate, continuously decreasing between 0 and 2500 cycles;
- the second stage (Stage II) with low constant wear rate (steady-state wear process).

## 4. Discussion

### 4.1. Methodology

The Hertzian theory of elastic contact between a sphere and a plane allows estimating the contact pressure under static conditions of loading, at the beginning of the test, before applying sliding. Considering that the order of magnitude of Young's modulus for both materials is 10 GPa [18], the calculated average contact pressure is *P*_avg_ ≈ 15 MPa and the maximum local contact pressure *P*_max⁡_ in the centre of the contact area is *P*_max⁡_ ≈ 23 MPa. These estimated values are lower by an order of magnitude to the compressive strength of the materials [7]. In regard to this argument, the 5 N load could be applied on the samples without producing cracks.

A mechanical property determining for the wear resistance of a material is its hardness. The higher the hardness is, the better the wear resistance is. The Archard's law expresses that the wear rate is inversely proportional to the hardness. In a preliminary study, the evolution of the Vickers microhardness was measured on both materials immersed in distilled water or Ringer's solution during 189 days after immersion. For each material, in a given medium (water or Ringer's solution), the evolution of the microhardness value was measured on 24 samples identical to those used for wear measurements. No significant statistical difference was found between the values determined for a given material in both media. After 28 days of immersion the microhardness values become constant. After 28 days, the following stable values were found (81 ± 12) HVN for C-GIC and (51 ± 12) HVN for RM-GIC. The obtained microhardness values are in agreement with those reported by several authors [14, 19–23].

### 4.2. Results

The microhardness measurements are an indicator of the maturity of the setting reaction [19, 24]. It has been previously reported that the immersion media have no incidence on the maturation process but have an influence on the microhardness values [17, 21] and tribological behaviour [13]. However, in the present study, each GIC result does not exhibit any significant difference between the wear data obtained in both solutions.

Unlike results reported for dental composite resin [5, 9], the wear rate of glass ionomer cement is not constant during the experiment: this result is in agreement with the observation of Yap et al. [19], but it does not follow the Archard's law which specifies that the wear is proportional to the sliding distance under constant applied normal force and sliding rate [12, 25]. In Figure 3, a linear increase of wear with time, or sliding distance, is only observed during Stage II. However, literature review provides many wear studies, in which the Archard's law is not verified throughout the wear test [26–31]. The main reason for this discrepancy is the existence of the so-called running-in period at the beginning of the tribological tests [31–33]. During this period, a change in the contact conditions between tribological surfaces occurs. This change is particularly important under conditions of nonconformal contact, such as the sphere-on-plane contact used in this work [27–31], and often leads to a transition between initial severe wear and further mild wear.

The evolution of wear with time reported in many studies carried out with pin-on-disc on ceramics as well as metals is similar to that presented in Figure 3. This type of evolution could be explained by different models [31, 34, 35] in which the wear rate decreases during the first running-in stage and tends to a constant value corresponding to a second stage where a steady-state wear process takes place. In this experiment, the running-in phase occurred before 2500 cycles and the steady-state wear phase is observed between 2500 and 7500 cycles.

During a sliding test performed under sphere-on-plane contact conditions, the contact area between the antagonists is the minimum at the beginning of the test. In the experimental conditions, according to the Hertzian contact theory, the applied normal force is low enough that the materials are only subjected to elastic deformation. However, this theory only applies to static contact conditions and to perfectly smooth surfaces. On real rough surfaces, during sliding, the tips of the asperities are subjected to compressive and shear stresses that can be much higher than those predicted by the Hertzian theory. Locally, the contact pressure can be much higher than the value leading to the rupture of the material. In these conditions, the material surface undergoes a severe wear process. Asperities are crushed or sheared and abrasive particles are generated, resulting in a high wear rate. As the number of cycles increase, the asperities are leveled, and if abrasive particles are removed from the contact at rate high enough, the surface is smoothed, increasing the real contact area. In addition, the width of the sliding track increases due to wear. As a result, the mean contact pressure decreases and the pressure distribution becomes more uniform. The reduction of the contact pressure is generally associated with a decrease in wear rate [27–29], which tends to a constant value corresponding to a stationary mild wear process.

Despite the fact that the Archard's law is not verified during the running-in period (Stage I) of the wear test, the highest wear rate and final worn volume at the end of this period are obtained with the RM-GIC, which has the lowest microhardness and which contains a photopolymerized resin that could interact with the acid-base setting process. The high initial wear rate decreases during this stage as a result of wear causing the increase of the contact area and the decrease of the contact pressure.

After the transition from running-in severe wear mechanism of Stage I to the mild wear mechanism of Stage II, the wear rates of the two materials become equal. This result could be explained by the fact that, during Stage II, under conditions of mild wear, the RM-GIC and the C-GIC have the same subsurface characteristics.

## 5. Conclusions

The immersion maturation media used have poor incidence on the tribological behaviour. The use of silicon replicas allows a dynamic nondestructive investigation and could be extended to other dental materials studies. Nevertheless, it should be interesting to study the superficial layer affected by sliding friction in Stage I on both materials as well as other parameters (the applied load and acidic or basic immersion media).

The studied materials have different early wear kinetics, but they have similar behaviors after 2500 cycles. Regarding the clinical point of view, efforts should be undertaken to improve this early wear by modifying the composition of the materials or by applying a protective layer. Coating materials already exist but have not been studied regarding their incidence on wear kinetics. These coating materials should be investigated both on maturation of the underlying material and on its wear behaviour.

## Figures and Tables

**Table 1:** Regression line equations and correlation coefficient *r*.

Maturation media	Material	Equation of the regression line	*r*
Ringer's solution	RM-GIC (Fuji II LC)	*y* = 33 · 10^−6^ *x* + 5.6 · 10^−1^	0.998
	C-GIC (Fuji IX GP)	*y* = 35 · 10^−6^ *x* + 2.3 · 10^−1^	0.999

Distilled water	RM-GIC (Fuji II LC)	*y* = 29 · 10^−6^ *x* + 4.8 · 10^−1^	0.994
	C-GIC (Fuji IX GP)	*y* = 38 · 10^−6^ *x* + 1.6 · 10^−1^	0.998

**Table 2:** Mean wear measurements.

Maturation media	Material	Mean wear between 2500 and 7500 cycles mm^3^/cycle (±SD)
Ringer's solution	RM-GIC (Fuji II LC)	33 · 10^−6^ ± 19 · 10^−6^
	C-GIC (Fuji IX GP)	35 · 10^−5^ ± 12 · 10^−6^

Distilled water	RM-GIC (Fuji II LC)	29 · 10^−6^ ± 9 · 10^−6^
	C-GIC (Fuji IX GP)	38 · 10^−6^ ± 29 · 10^−6^
